# Er-Bai-Tang decoction improved the movement disorders and neuronal injury in the Parkinson’s disease model rats via decreasing p38 MAPK pathway and improving the composition of intestinal flora

**DOI:** 10.1590/acb371104

**Published:** 2023-01-06

**Authors:** Jinrong Li, Yuehan Ni, Li Huang, Xinyuan Yu, Jianwei Zhu

**Affiliations:** 1 MS. Chongqing Traditional Chinese Medicine Hospital – Chongqing, China.; 2 MS. Yuyao Traditional Chinese Medicine Hospital – Yuyao, China.; 3 St. Chongqing Traditional Chinese Medicine Hospital – Chongqing, China.; 4 PhD. Chongqing Traditional Chinese Medicine Hospital – Chongqing, China.

**Keywords:** Parkinson Disease, p38 Mitogen-Activated Protein Kinases, Gastrointestinal Microbiome

## Abstract

**Purpose:**

Our previous study showed that Er-Bai-Tang decoction (EBT) could effectively improve Parkinson’s disease (PD) patients’ quality of life, sleep, mood, and cognitive disorders, but the mechanism of EBT to treat PD was unclear. So, our study aimed to explore the mechanism of EBT to treat PD via p38 mitogen-activated protein kinases (MAPK) pathway and intestinal flora.

**Methods:**

In our study, the PD rat model was established by subcutaneously injecting 2 mg/kg/d rotenone solution, and 23.43 g/kgEBT was used to treat PD model rats.

**Results:**

Behavioral test showed that EBT could reverse the motor impairment in the PD model rats. Hematoxylin and eosin result showed that EBT could reduce the cell necrosis in the SNpc area of the PD model rats. Western blotting and real time-polymerase chain reaction showed that EBT could decrease the p38 MAPK expression in the SNpc area of the PD model rats. 16s rRNA sequencing analysis showed that EBT could improve the composition of intestinal flora in the PD model rats. Rikenellaceae at family level and *Alistipes* and *Allobaculum* at the genus level were the key species in the PD development and EBT treatment to PD. KEGG showed that EBT might change the iron uptake in PD rats.

**Conclusions:**

EBT could improve the motor symptoms and neuronal injury in the PD model rat, and its mechanism may be related to decreasing p38 MAPK pathway and improving the composition of intestinal flora.

## Introduction

Parkinson’s disease (PD) is an age-related debilitating neurodegenerative disorder characterized pathologically by selective loss of dopaminergic neurons in the substantia nigra pars compacta (SNpc) accompanied by a decrease in dopamine (DA) level, and intracytoplasmic Lewy bodies aggregated by α-synuclein (α-Syn)^1^. The progressive loss of midbrain dopamine neurons is the leading cause of motor dysfunction in PD^2^^-^ 4. The current mainstay therapy for PD is dopamine replacement. However, long-term dopamine replacement can induce severe adverse effects, such as motor response fluctuations and dyskinetic movements^5^^-^ 7. Therefore, it is necessary to explore new treatments against PD.

It has been reported that p38 mitogen-activated protein kinases (MAPK) was activated in DA neurons in the SNpc in PD patient samples and PD mouse models^8^^,^ 9. The active p38 MAPK promotes the nuclear localization of p53 and the phosphorylation of Parkin-1 and controls oxidative and inflammatory stress in DA neurons, causing DA neuron loss^10^^-^ 12. Inhibiting p38 MAPK activation could reduce DA neuron loss in PD^13^^,^ 14, showing p38 MAPK is a key pathway for PD treatment^15^.

Many studies showed that intestinal flora performed an important regulation in the development and treatment of PD, and microbiome modulation may benefit PD patient^16^^,^ 17. Intestinal flora is also a therapeutic target for PD.

Our previous study showed that Er-Bai-Tang decoction (EBT) could effectively improve PD patients’ quality of life, sleep, mood, and cognitive disorders^18^, but the mechanism of EBT to treat PD was unclear. Exploring the role and mechanism of traditional Chinese medicine through the gut microflora has become a research hotspot in the treatment of disease. Therefore, our study aimed to explore the mechanism of EBT to treat PD via p38 MAPK pathway and intestinal flora.

## Methods

### Establishment of Parkinson’s disease rat model and treatment

Forty-five specific pathogen free Sprague Dawley rats (body weight 200 ± 20 g) were randomly divided into three groups: control group, model group, and EBT group, with 15 rats in each group. Rats in model group and EBT group were subcutaneously injected with 2 mg/kg/d rotenone solution (soluble in sunflower oil) through the back of the neck for 28 days to establish the PD model rats. The rats in the control group were subcutaneously injected the same volume of sunflower oil once a day, for consecutive 28 days. After the successful establishment of PD rat model, the rats in the control group and model group were intragastric with normal saline, and EBT group was intragastric with EBT decoction. The dosage of EBT decoction was: 225 g (crude drug) / 60 kg × 6.25 = 23.43 g/kg, decocted into 0.78 g/mL, gavage 3 mL each time, twice a day, for consecutive 30 days.

### Behavioral test

Feces samples of rats in each group were collected the next day after the last administration, and the behavior of rats in each group was evaluated.

### The open field test

After being placed in the center grid, firstly, the rats were adapted to the environment for 10 min, and then the autonomous activities of the rats within 5 min were recorded. The movement time and horizontal movement distance of the rats were counted.

### The pole climbing test

A cork ball with a diameter of 25 cm was fixed on the top of a wooden pole with length of 50 cm and diameter of 1 cm, and gauze was wrapped around the pole to prevent slipping. Then, the tested rats were placed on the ball, and the time needed for the rats to get off the ball was recorded. Then, score according to the following standards: 3 points for completing the action within 3 seconds; 2 points for completing the action within 6 seconds; 3 points for completing the action than 6 s. Finally, the score was calculated.

### The tail suspension test

The rats were suspended on a horizontal wire. The rats were given a score of 3 for grasping the wire with both hind paws, 2 for grasping the wire with one hind paw, and 1 for not grasping the wire with either hind paw.

### The force swimming test

The rats were put into a 20 cm × 30 cm × 20 cm water tank for 5 min, and the water temperature was 22 ~ 25 °C. The scoring criteria were as follows: the rats were given a score of 3 for swimming continuously during the test time, 2.5 for swimming most time during the test time, 2 for floating time more than 50% of the whole test time, 1.5 for occasionally swimming and 1 for hardly ever swimming. Finally, the score was calculated.

### Sample collection

After behavioral test, the rats were weighed and anesthetized by 4% pentobarbital sodium (40 mg/kg). Then, the rats were sacrificed. The SNpc tissues of rats were taken out, some of them were fixed with 4% formaldehyde, and some of them were frozen in liquid nitrogen.

### Hematoxylin and eosin staining

The SNpc tissues of rats were dehydrated by alcohol, transparent by xylene, deparaffinized, rehydrated and sliced. The sections were ewaxed by xylene, dehydrated by ethanol, stained by hematoxylin and eosin (HE), and finally sealed by neutral gum. The pathological changes were observed.

### Real time-polymerase chain reaction

Total RNA was extracted from the SNpc tissues of rats, and reverse transcription was performed. After amplification, real time-polymerase chain reaction (qRT-PCR) reaction was performed to detect P38 MAPK mRNA expression. β-actin was used as internal reference.

### Western blot

Total protein was extracted from the SNpc tissues of rats and quantitated using a bicinchoninic acid (BCA) assay. Proteins were separated with SDS-PAGE and transferred to a polyvinylidene difluoride (PVDF) membrane. The membrane was blocked by 5% nonfat dry milk for 1 h and incubated with primary antibody (anti-p38 MAPK, diluted 1:1,000) over night. Then, the respective secondary antibody was incubated for 1 h at room temperature. Densitometric analysis was performed using a Bio-Rad image acquisition system (Bio-Rad Laboratories). β-Actin was used as an internal reference to detect the relative expression of p38 MAPK protein.

### 16s rRNA sequencing analysis

The 16s rRNA sequencing work of rat feces was performed by Shanghai Paisennuo Biotechnology Co. LTD. Major steps include the following: total microbial DNA was extracted from fecal samples using stool DNA extraction kit. DNA was purified and quantified by quantitative qRT-PCR. Then, the paired-end sequencing on an Illumina MiSeq sequencer was performed. The QIIME software was used to process the sequencing data, and microbial composition analysis and metabolic pathway statistics were analyzed by the KEGG Pathway database (http://www.genome.jp/kegg/pathway.html).

### Data analysis

All data are expressed as mean ± standard deviation. For comparisons between multiple groups, data were analyzed by one-way analysis of variance (ANOVA), and Tukey’s post hoc test was performed to compare data of multiple groups. P < 0.05 was considered to show significant difference.

## Results

### Er-Bai-Tang decoction treatment reversed the motor impairment in the Parkinson’s disease model rats

**Figure 1:**
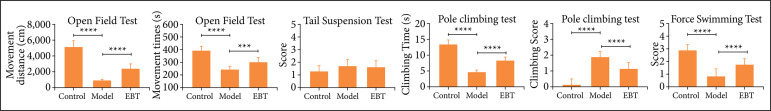
Behavioral results.

Behavioral results included the open field test, pole climbing test, tail suspension test, and force swimming test (Fig. 1). Compared to the control rats, the PD model rats showed the poorest motor ability, and EBT treatment reversed the motor impairment in the PD model rats.

EBT: Er-Bai-Tang; ***P < 0.001; ****P < 0.0001.

### Er-Bai-Tang treatment reduced the cell necrosis in the SNpc area of the Parkinson’s disease model rats

**Figure 2:**
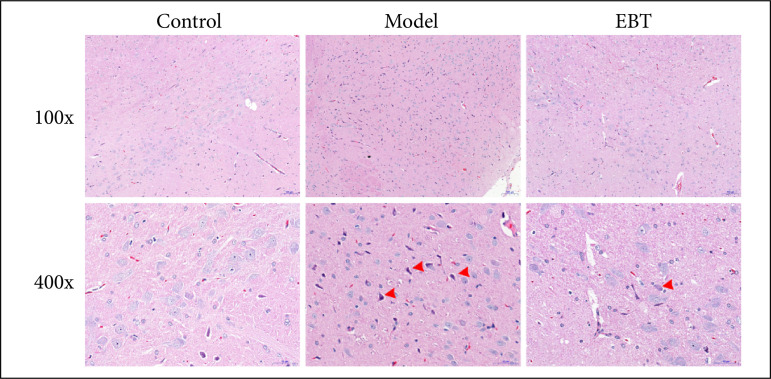
Hematoxylin and eosin results of the SNpc area.

The HE results are shown in Fig. 2. Compared to the control rats, more necrosis was found in the SNpc area in the PD model rats, and EBT treatment reduced the cell necrosis in the SNpc area of the PD model rats.

EBT: Er-Bai-Tang; red arrow: necrosis.

### Er-Bai-Tang treatment inhibited the increased expression of p38 MAPK in the Parkinson’s disease model rats

**Figure 3:**
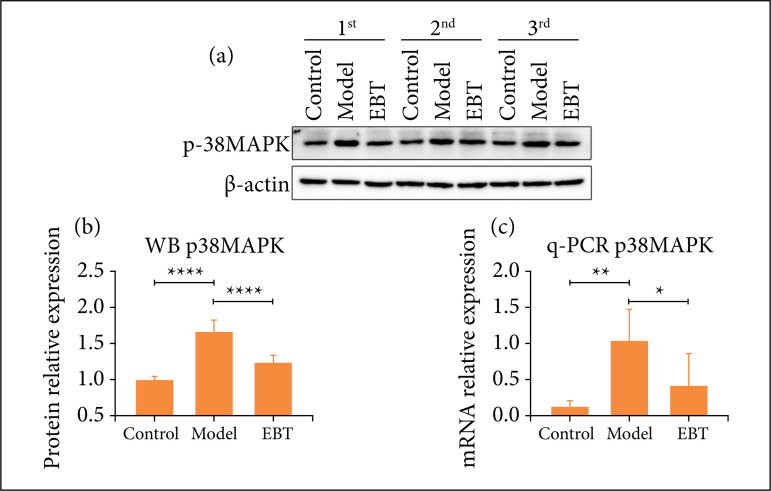
WB and q-PCR detected the expression of p38 MAPK in the SNpc area. (**a-b**). WB results for p38 MAPK. **(c)** q-PCR results for p38 MAPK.

EBT: Er-Bai-Tang; MAPK: mitogen-activated protein kinases; WB: Western blotting; q-PCR: real time-polymerase chain reaction; *P < 0.05; **P < 0.01; ****P < 0.0001.

In this part, we detected the expression of p38 MAPK in the SNpc area. As shown in Figs. 3a-3c, the PD model rats increased the expression of p38 MAPK mRNA and protein, and EBT treatment inhibited the increased expression of p38 MAPK mRNA and protein in the PD model rats.

### Er-Bai-Tang could improve the composition of intestinal flora in the Parkinson’s disease model rats

**Figure 4:**
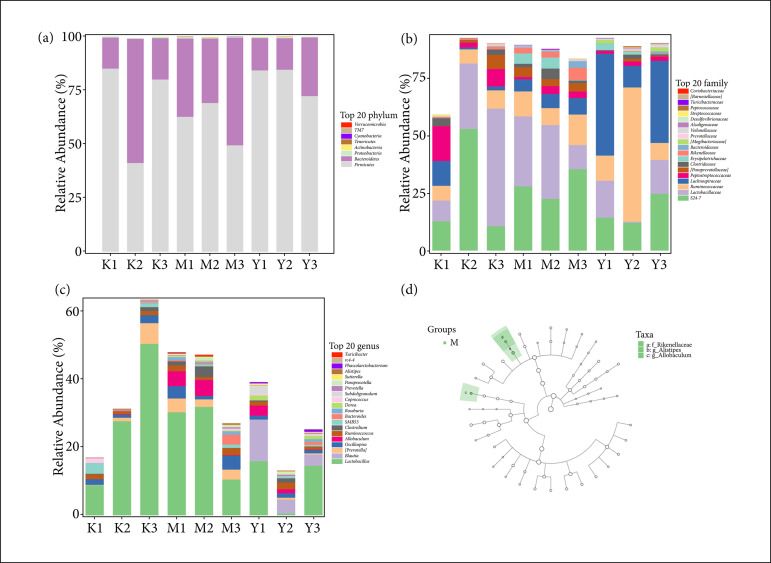
The composition of intestinal flora in the rats. The intestinal flora composition at **(a)** phylum, **(b)** family, and **(c)** genus levels. **(d)** LEfse analysis was performed to reveal the significant of abundant modules. Hollow nodes represent taxa with no significant difference among three groups, while green node indicates that these taxa show significant difference among three groups and are more abundant in the model group.

To clarify the effect of EBT on intestinal flora in the PD rats, we used 16S rRNA gene sequencing technology to analyze the changes in intestinal flora. The intestinal flora composition at phylum, family, and genus levels in the control, model, and EBT groups is showed in Figs. 4a-4c. Next, LEfse analysis was performed to reveal the significant of abundant modules among the three groups. As shown in Fig. 4d, at phylum level, no significant change in intestinal flora was observed among the three groups; At family level, Rikenellaceae was the key species and significantly increased in the PD model groups. At the genus level, *Alistipes* and *Allobaculum* were the key species and also significantly increased in the PD model rats. These suggested that EBT could improve the composition of intestinal flora in the PD model rats.

K: control; M: model; Y: EBT: Er-Bai-Tang.

### Er-Bai-Tang could change the metabolic pathways of intestinal flora in the Parkinson’s disease model rats

**Figure 5:**
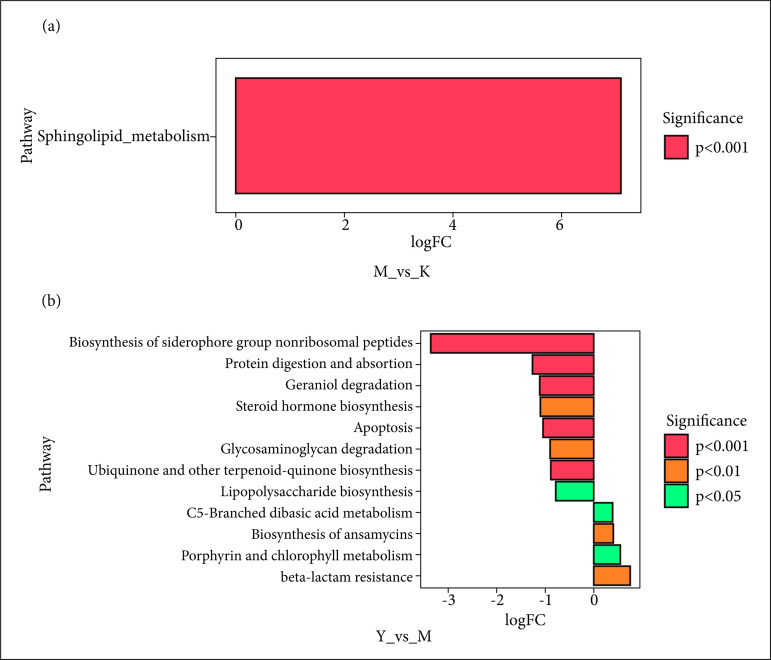
KEGG analysis of intestinal flora in the rats. **(a)** The differential metabolic pathways between model **(M)** group and control **(K)** group. **(b)** The differential metabolic pathways between Er-Bai-Tang **(T)** group and model **(M)** group.

KEGG analysis was used to explore the differential metabolic pathways among the three groups. As shown in Fig. 5a, the PD model rats significantly increased the Sphingolipid metabolism of intestinal flora compared to the control rats. As shown in Fig. 5b, compared to PD model rats, EBT treatment mainly inhibited the biosynthesis of siderophore group nonribosomal peptides of intestinal flora in the PD model rats. These suggested that EBT could change the metabolic pathways of intestinal flora in the PD model rats.

## Discussion

The loss of substantial nigral projections neurons, which results in DA levels in the dorsal striatum, is the primary cause of PD. As a precursor for DA, l-3,4-dihydroxyphenylalanine or levodopa (l-DOPA) effectively alleviates motor symptoms in PD. However, long-term use of l-DOPA induced dyskinesia^19^. In our study, EBT alleviated motor impairment and reduced cell necrosis in the SNpc area in the PD rats, suggesting that BET might be a potential therapeutic agent for PD.

Some investigations demonstrated that the p38 MAPK pathway was a therapeutic target of Chinese herbal medicine in PD. For example, calycosin attenuates 1-Methy-l-4-phenyl-1,2,3,6-tetrahydropyridine (MPTP)-induced PD by inhibiting the activation of TLR/NF-κB and MAPK pathways^20^; polyphenols from *Toona sinensis* seeds alleviate neuroinflammation induced by 6-hydroxydopamine through down-regulating p38 MAPK signaling pathway in PD rats^21^. Interestingly, *Atractylodis Rhizoma* Alba and *Bupleurum* L., Chinese herbal ingredients in EBT, were both found anti-inflammation *in vitro* via suppressing p38 MAPK signaling pathway^22^^,^ 23. In our study, we also observed that EBT inhibited p38 MAPK signaling pathway in the SNpc area of PD rats and performed a protective role in PD development.

Some evidence supports the potential roles of gut microbiota in PD^24^^,^ 25. Lorente-Picón and Laguna summarize that^25^: at the phylum level, the levels of Proteobacteria, Actinobacteria, and Verrucomicrobia were increased in PD; at the family level, the relative abundances of Prevotellaceace and Lachnospiraceae were reduced and the ones of Lactobacillaceae, Bifidobacteriaceae, Enterobacteriaceae, and Verrucomicrobiaceae were increased in PD; at the genus level, the relative abundances of *Blautia*, *Roseburia* and *Faecalibacterium* were decreased and the ones of *Lactobacillus* and *Akkermansia* were increased in PD. In our study, we found that: Rikenellaceae at family level and *Alistipes* and *Allobaculum* at the genus level were the key species in the PD development and EBT treatment to PD. EBT inhibited the increase of that gut microbiota in PD, suggesting that EBT could improve the composition of gut microbiota in the PD model rats.

Finally, the potential functions of the gut microbiota in PD rats under EBT treatment were predicted using the KEGG database, and our study showed that EBT treatment mainly inhibited the biosynthesis of siderophore group nonribosomal peptides of intestinal flora in the PD model rats. Siderophore group nonribosomal peptides are natural products commonly produced by bacteria and fungi^26^^,^ 27 and have essential effects on the host’s life. For example, Bacillibactin, one nonribosomal peptide discovered from *Bacillus subtilis*, serves as a catecholic siderophore to regulate iron uptake^28^. Therefore, EBT might change the iron uptake in PD rats.

Our study showed that EBT could reverse the motor impairment and reduce the neurons necrosis in the SNpc area in the PD model rats, and its mechanism may be related to decreasing p38 MAPK pathway and improving the composition of intestinal flora. Rikenellaceae at family level and *Alistipes* and *Allobaculum* at the genus level were the key species in the PD development and EBT treatment to PD. KEGG showed that EBT might change the iron uptake in PD rats. Our study provides strong evidence of a beneficial role of the EBT on PD.

## Conclusion

Our study showed that EBT could reverse the motor impairment and reduce the neurons necrosis in the SNpc area in the PD model rats and its mechanism may be related to decreasing p38 MAPK pathway and improving the composition of intestinal flora. Rikenellaceae at family level and Alistipes and Allobaculum at the genus level were the key species in the PD development and EBT treatment to PD. KEGG showed that EBT might change the iron uptake in PD rats. Our study provides strong evidence of a beneficial role of the EBT on PD.
